# 

**Published:** 1900-05-19

**Authors:** 


CADBURY'S COCOA NO DRUGS OR ALKALIES USED.

No. 712. Vol. XXVIII. [Registered as a Newspaper.] SATURDAY, MAY 19, 1900. [Subscription Terms, see p. 114.] PRICE TWOPENCE.

